# The colorectal liver metastasis growth pattern phenotype is not dependent on genotype

**DOI:** 10.1038/s41416-025-03103-4

**Published:** 2025-07-23

**Authors:** Diederik J. Höppener, Sanne M. L. Verheul, Pieter M. H. Nierop, Florian E. Buisman, Boris Galjart, Saskia M. Wilting, Siân A. Pugh, Susan D. Richman, Vinod P. Balachandran, William R. Jarnagin, T. Peter Kingham, Peter B. Vermeulen, Jinru Shia, Philip Quirke, John A. Bridgewater, Timothy S. Maughan, Bas Groot Koerkamp, Dirk J. Grünhagen, Cornelis Verhoef, John N. Primrose, Michael I. D’Angelica

**Affiliations:** 1 Departments of Surgical Oncology and Gastrointestinal Surgery, Erasmus MC Cancer Institute, Rotterdam, the Netherlands; 2 Medical Oncology, Erasmus MC Cancer Institute, Rotterdam, the Netherlands; 3 Department of Oncology, Addenbrooke’s Hospital, Cambridge, UK; 4 Leeds Institute of Medical Research at St James’s, St James’s University Hospital, University of Leeds, Leeds, UK; 5 Departments of Surgery, Memorial Sloan Kettering Cancer Center, New York, NY USA; 6 Translational Cancer Research Unit, ZAS Augustinus, Antwerp, Belgium; 7 Pathology, Memorial Sloan Kettering Cancer Center, New York, NY USA; 8 UCL Cancer Institute, University College London, London, UK; 9 MRC Oxford Institute for Radiation Oncology, University of Oxford, Oxford, UK; 10 Department of Surgery, Erasmus MC, Rotterdam, the Netherlands; 11 Department of Surgery, University of Southampton, Southampton, UK

**Keywords:** Cancer genetics, Colorectal cancer, Cancer genomics, Metastasis, Tumour heterogeneity

## Abstract

**Background:**

The histopathological growth patterns (HGPs) of colorectal cancer liver metastases broadly classify patients into two groups post-liver metastasectomy, with encapsulated HGP indicating a more favourable prognosis. The potential association between HGPs and specific mutations is poorly understood.

**Methods:**

Using next-generation sequencing data of 461 resected patients (104 patients with encapsulated versus 357 patients with non-encapsulated HGP), 19 putative colorectal cancer driver genes, tumour mutational burden (TMB), and microsatellite instability (MSI) or *POLE* mediated hypermutation were compared.

**Results:**

Most putative drivers, including *KRAS* (*q* = 0.89), *NRAS* (*q* = 0.98),) and *BRAF* (*q* = 0.97)), were not associated with HGP. However, mutations in *B2M* and *PTEN* were associated with a encapsulated phenotype (7% vs. 0%, *q* = 0.001, and 9% vs. 2%, *q* = 0.02, respectively). TMB was higher in encapsulated patients (median 5.8 vs. 5.1 mutations per megabase, *p* = 0.009). Multivariable overall survival analysis corrected for genetic and patient factors confirmed that the encapsulated phenotype was an independent prognostic factor (adjusted hazard ratio, 0.60; 95% confidence interval: 0.36–0.99). Upon stratified analysis, all identified genetic associations were equivocal between the cohorts.

**Conclusions:**

While an association between genetic drivers of adaptive immune responses seems probable and could explain a minority of encapsulated patients, these results primarily demonstrate that HGP phenotype is independent of the tumour genotype.

## Introduction

Colorectal adenocarcinoma, the third cause of cancer-related mortality worldwide, can be divided into two genetically distinct types [1, 2]. The majority of colorectal cancers exhibit chromosomal instability, while a smaller subset remains chromosomally stable but are hypermutated. Hypermutated tumours are commonly related to mutations in genes encoding mismatch/proofreading activity, such as DNA polymerase epsilon (POLE), and can lead to microsatellite instability [1, 3]. Metastatic spread often occurs, with the liver affected most frequently [4].

Immunogenicity, prognosis, and genomics are interrelated [5]. For metastatic colorectal cancer, *KRAS*, *NRAS*, and *BRAF* mutations are prognostically unfavourable and help select patients for EGFR inhibition [6]. In contrast, the hypermutated forms of colorectal cancer are associated with immunogenic tumours, and are known to metastasise less frequently [7, 8]. Additional evidence for genomics as a driver of anti-cancer immune responses arises from the treatment with immune checkpoint inhibitors, from which highly mutated cancers, such as melanoma and lung adenocarcinoma, benefit most [9, 10]. Compared to other solid tumours, metastatic colorectal cancer is below average in mutational load [11], and a benefit of immune checkpoint blockade therapy has yet to be demonstrated beyond the minority (<4%) of hypermutated microsatellite instable tumours [12].

Histological phenotyping of liver metastases allows for the identification of a favourable metastatic colorectal cancer subtype by recognising distinct histopathological growth patterns at the tumour-liver interface [13, 14]. In the so-called encapsulated growth pattern, a fibrotic capsule borders the entire periphery, barring cell-to-cell contact between the liver and tumour [13]. The encapsulated metastases are angiogenic and inflamed, with a microenvironment enriched for both T and B cells [15–19]. The non-encapsulated metastases display an infiltrative growth pattern marked by tumour cell-hepatocyte contact, exhibit vessel co-option, and are immunologically scarce [13, 15–19]. The one-fifth of patients with the encapsulated phenotype exhibit remarkably good prognosis for metastatic colorectal cancer, achieving a five-year overall survival after metastasectomy of 80% compared to 40% in the non-encapsulated phenotype after metastasectomy [20, 21]. Differences in tumour genomic between the growth pattern phenotypes are currently lacking.

By comparing tumour mutational burden (TMB), the incidence of hypermutated tumours, and driver gene mutations, this study sought to identify potential genetic alterations related to growth pattern phenotypes in liver metastatic colorectal cancer.

## Methods

### Patient cohorts

Patients were selected who underwent the first resection of colorectal liver metastasis at Memorial Sloan Kettering Cancer Center (MSKCC) until January 2019 and for whom the growth pattern could be determined on that resection specimen. Next-generation sequencing had to be performed on the resection or biopsy specimen of the primary or liver metastasis colorectal adenocarcinoma, prior to or within one year following liver metastasis surgery. Sequencing was performed using the MSK-IMPACT assay in the clinical laboratories of the Molecular Diagnostics Service of MSKCC. All the patients signed a clinical (IRB #16-1343 or #15-044) or research (IRB #12-245) consent form for genomic sequencing [22]. Median follow up time was 24 months, patients lost follow up were censored and concerning missing data no imputation was used only complete analyses were performed.

The cohort was expanded using patients from the New EPOC phase III prospective randomised controlled trial [23]. This trial randomly allocated 257 *KRAS* exon-2 (codons 12, 13, and 61) wild-type patients with resectable and suboptimal resectable colorectal cancer liver metastasis to an intended 12 weeks of pre- and postoperative systemic chemotherapy (CAPOX, FOLFOX, or FOLFIRI) with or without cetuximab, an anti-epidermal growth factor receptor (EGFR) antibody. The trial was stopped at interim analysis because of worse progression-free survival in the experimental cetuximab arm [23]. The short- and long-term results have since been published [23, 24]. For 233 subjects, next-generation sequencing of the primary tumour and/or liver metastasis specimen(s) was performed by the S:CORT consortium at the Wellcome Sanger Institute. Those with available sequencing data and digitalised hematoxylin and eosin (H&E)-stained slides of resected liver metastasis on which the growth pattern phenotype could be determined were selected. All patients provided written informed consent for further research on their samples during trial enrolment. Median follow up time was 69 months, patients lost follow up were censored and concerning missing data no imputation was used only complete analyses were performed.

Patient data on sex, age at liver resection, primary colorectal adenocarcinoma characteristics including location and TNM stage, number and size of liver metastases, preoperative serum carcinoembryonic antigen (CEA) levels, time between primary tumour treatment and diagnosis of metastatic disease, treatment details including perioperative systemic chemotherapy, and survival were extracted from either the prospectively maintained institutional database or the collated clinical trial data.

### Histopathological classification

All available/digitalised H&E-stained tissue slides of resected colorectal liver metastasis specimens of eligible patients were reviewed by light microscopy at the pathology department of MSKCC or digitally for the New EPOC patients. The histopathological growth patterns were assessed by two trained observers simultaneously and in accordance with international consensus guidelines, which provide in-depth details of the assessment algorithm [13]. Excellent interobserver agreement has been shown between trained observers and expert pathologists [25]. Upon histopathological assessment the border between the liver metastasis and the liver parenchyma was systematically reviewed for growth pattern phenotype. If this border exclusively showed a capsule of fibrous tissue separating the liver metastasis from the liver parenchyma in all resected tumours, the patient was classified as encapsulated (Supplementary Fig. 1A–C). Otherwise, if tumour cells were seen directly pushing against or in continuum with the liver cell plates at any part of (Supplementary Fig. 1D–F) or throughout the entire border (Supplementary Fig. 1G–I) in any or all resected tumour(s), the patient was classified as non-encapsulated (Supplementary Fig. 1D–I). This classification is classified as encapsulated versus non-encapsulated, which is most relevant from a clinical point of view [20, 21], as also recognised by the updated consensus statement [14]. While this classification may appear prone to sampling error, excellent intra- and intermetastasis concordance of 95% and 90% exists [25]. All growth pattern assessments were performed blinded for patient characteristics and sequencing results.

### Next generation sequencing

For MSKCC patients, the Memorial Sloan Kettering-Integrated Mutation Profiling of Actionable Cancer Targets hybridisation capture-based next generation sequencing assay, or MSK-IMPACT, was used. This custom designed sequencing panel has been expanded over time to include 341, to 410, and more recently 468 genes. For this study, only samples sequenced with the 410 or 468 IMPACT panel (Supplementary Table 1) were considered, based on the capture of driver genes of interest (see *driver genes*). Targeted sequencing was performed using custom-designed DNA probes of all exons and selected introns of the panel genes [26]. Matched tumour and normal DNA were extracted from formalin-fixed paraffin-embedded (FFPE) primary colorectal adenocarcinoma and/or liver metastasis tissue and patient blood, as previously described [22, 26]. In case both the primary and metastatic samples were sequenced, only the latter was included in the analysis. Sequencing to high, uniform coverage (median coverage >500x) was performed using an Illumina HiSeq 2500 system. Genomic alterations, including single- (SNV) and multi-nucleotide variants (MNV), and insertions and deletions (indels), were determined and called against the matched normal sample. H&E-stained slides were reviewed for all sequenced samples by pathologists to confirm the diagnosis of colorectal adenocarcinoma.

For New EPOC patients, 80 colorectal cancer driver genes (Supplementary Table 1) hybridisation DNA target capture panel (SureSelect, Agilent) spanning all coding exons was used, followed by next-generation sequencing on Illumina systems to achieve high, uniform coverage. DNA was extracted from archival FFPE tissue of primary colorectal adenocarcinoma and/or liver metastasis tissue using an adjacent H&E slide for confirmation of tumorous matter and microdissection, as previously described [27]. In case both the primary and metastatic samples were sequenced, only the latter was included in the analysis. Variant calling for SNV and indels was performed using Caveman and Pindel software, respectively.

### Driver genes

The associations of previously classified high-confidence (metastatic) colorectal cancer driver genes with the growth pattern phenotype were investigated. The selection was based on Bailey et al. [28], who identified 20 colorectal adenocarcinoma driver genes using a comprehensive pan-cancer approach in 9,079 samples of 33 tumour types from The Cancer Genome Atlas (TCGA), and Mendelaar et al. [7], who identified 23 driver genes in 429 tumour samples of metastatic colorectal cancer patients within the pan-cancer CPCT-02 study. Among these 15 genes overlapped, resulting in a total of 28 driver genes, 19 of which were targeted in the 410 and 468 IMPACT panels, as well as in the 80 gene panel used in the New EPOC: *APC*, *ARID1A*, *ATM*, *B2M*, *BRAF*, *CTNNB1, FBXW7, GNAS, KRAS, NRAS, PIK3CA, PTEN, RNF43, SMAD2, SMAD3, SMAD4, SOX9, TCFL2, TP53*. The number of genetic alterations in the selection of 19 colorectal driver genes was compared between the growth pattern phenotypes. For *BRAF* only V600E mutations were included. For all other genes, the selection of driver versus passenger mutations was based on the OncoKB database, and only known or suspected oncogenic variants were considered.

### Signalling pathways

Pathway analysis was performed to evaluate whether any mutations existed in the driver genes belonging to the Wnt/β-catenin (*APC, CTNNB1, RNF43* and *TCF7L2*), MAPK (*BRAF, KRAS*, and *NRAS*), TGF-β (*SMAD2, SMAD3* and *SMAD4*), and/or PI3K (*PTEN* and *PIK3CA*) pathway(s). Alterations were visualised in oncoplots stratified for histopathological growth patterns using the ComplexHeatmap package (v.2.5.5).

### Mutational load and hypermutation

TMB was calculated for all patients by dividing the number of coding mutations (i.e., all SNV, MNV, and indels) by the total genetic target region captured in each panel, which was 1.38, 1.53, and 0.66 megabases (Mb) for the 410 and 468 IMPACT, and 80 gene New EPOC panels, respectively [22]. Such estimates of TMB using targeted next generation sequencing can exhibit strong correlation with results from whole exome sequencing, although these are dependent on the respective panel size and gene selection [29]. In accordance with literature, samples were classified as hypermutated in case of >12 mutations/Mb [1]. Mutational load and hypermutation were compared by growth pattern phenotype. Separate analyses were performed, excluding MSI-H- and *POLE* mediated hypermutations.

### Microsatellite instability and POLE mediated hypermutation

Both microsatellite instability-high (MSI-H) and pathogenic *POLE* mutation-related hypermutated forms of colorectal cancer were compared based on the growth pattern phenotype.

All mutational variants of the DNA polymerase epsilon (*POLE*) proofreading domain identified in both cohorts were assessed for pathogenicity using the OncoKB database and the literature [3].

MSK-IMPACT incorporates the MSIsensor score to determine microsatellite instability (MSI) based on next-generation sequencing data [30]. The MSIsensor calculates the percentage of unstable microsatellites among all microsatellites tested. Tumours with a percentage of ≥10 were considered MSI-H, and others microsatellite stable (MSS) [30]. MSIsensor has proven reliable, with a reported 98.6% concordance rate between MSIsensor and immunohistochemistry [30]. For New EPOC patients, MSI status was also determined based on next generation sequencing data using a total of 123 MSI markers included within the panel. Those with >2 mutations were classified as MSI-H, and others as MSS [27].

### Co-occurrence and mutual exclusivity

Co-occurrence and mutual exclusivity between driver genes was assessed using the Discrete Independence Statistic Controlling for Observations with Varying Event Raters, or DISCOVER test (v.0.9.3) [31]. Unlike many other tests, DISCOVER does not assume identical gene alteration probabilities across samples, making it more sensitive and better at controlling its false positive rate [31]. Mutual exclusivity and co-occurrence was assessed for patients with a encapsulated and non-encapsulated phenotype separately, and in patients with growth pattern as stratification factor. The relative percentage of co-occurring mutations with individual genes and the double mutation rates for all pairs were visualised for encapsulated and non-encapsulated patients separately in multilayer circular plots using the circlize (v.0.4.11) package [32].

### Statistics

Frequencies are reported as absolute counts with corresponding percentages, and non-parametric numerical data as medians with interquartile ranges (IQR). Categorical differences were inferred using the χ^2^ test and non-parametric data were compared using the Mann–Whitney U test. For multiple testing of mutation rate differences, correction according to Benjamini and Hochberg was applied, and q-values were reported. Statistically significant differences in gene mutation rates were assessed in multivariable logistic regression models, excluding MSI-H and *POLE* mediated hypermutation and correcting for TMB and cohort. Multivariable linear regression was performed to assess TMB and the number of driver gene mutations, correcting for MSI-H and *POLE* mediated hypermutations, cohort, and sample origin. Overall survival was compared using Kaplan–Meier analysis and multivariable Cox proportional hazards regression, with clinical risk factors (cohort, node-positivity, extrahepatic disease, >1 metastasis), genetic risk factors (hypermutation, *APC, TP53, KRAS, NRAS, BRAF*), and any identified genes associated with the growth pattern phenotype as covariates. Results from multivariable regression models were reported as odds ratios (OR), β-coefficients, or hazard ratios (HR) with corresponding 95% confidence intervals (95%CI) for logistic, linear, and survival regressions, respectively. Kaplan–Meier survival estimates were compared using the log-rank test, and the median follow-up for survivors was determined using the reverse Kaplan Meier method. Additional stratified genomic analyses were performed to assess whether the identified associations were consistent across cohorts. Statistical significance was defined as two-sided α < 0.05. Analysis and data visualisation were performed using the R Project for Statistical Computing version 4.1.0 (https://www.r-project.org/) with the packages previously mentioned, ggplot2 (v.3.3.2), rms (v.6.0-1), survival (v.3.2-7), survminer (v.0.4.8), and tableone (v.0.12.0).

## Results

Between 2014 and January 2019, 589 patients were surgically treated for colorectal liver metastasis at MSKCC, and a primary and/or colorectal adenocarcinoma liver metastasis specimen was sequenced using MSK-IMPACT. For 196 (33%) patients, sequencing was performed more than one year after liver surgery; therefore, these patients were not included. Targeted sequencing with a 410 or 468 gene panel was available for 362 of the 393 (92%) remaining patients. Upon histopathological examination, the growth pattern phenotype was able to be determined in 308 (85%) patients, with median (IQR) 2 (1-4) hepatic tumour(s) and 5 (3-8) H&E slides examined. Determination was not possible in 15 patients (4%) due to the absence of a resection specimen, in 12 patients (3%) the tumour was absent (e.g., complete pathological response), and for 27 patients (7%), H&E slides were missing.

Of the 233 New EPOC patients who had colorectal cancer samples sequenced, 160 (69%) had digitalised H&E sections of resected liver metastases available for assessment. Of the remaining patients, 26 (11%) did not undergo local liver metastasis treatment; in eight patients (3%), no residual tumour was reported on pathological examination of the hepatectomy specimen, and in 39 patients (17%), digital H&E slides were not available. The growth pattern was determined in 153 (96%) patients, with median (range) 1 (1-2) hepatic tumour(s) and 1 (1-2) slides examined. For seven patients (4%), assessment was not possible based on an absent or limited tumour-liver interface.

Patient, treatment, and sequencing characteristics of all 461 patients and comparisons for the cohort are reported in Table 1. A encapsulated phenotype was observed in 62 (20%) patients of MSKCC and 42 (27%) of New EPOC (*p* = 0.08, Table 1). Apart from age, which was significantly lower for MSKCC patients (*p* < 0.001), New EPOC patients had more favourable characteristics with fewer and smaller liver metastases (both *p* < 0.001), fewer right-sided primary tumours and extrahepatic disease (*p* = 0.06 and *p* < 0.001), and a longer disease-free interval (*p* < 0.001) (Table 1). More CRC samples were included in the MSKCC than in New EPOC (49% vs. 7%, *p* < 0.001). The average coverage depth was significantly higher in the MSKCC group, with a median (IQR) of 714x (594-844) versus 586x (477-654) (*p* < 0.001). The difference in sequencing panels translated into a significantly higher TMB (median [IQR] mutations/Mb) in the New EPOC cohort (6.1 [4.6–9.1]) than in the MSKCC cohort (5.1 [3.6–6.5]) (*p* < 0.001).

**Table 1:** Patient baseline, treatment, and sequencing characteristics by cohort

			MSKCC	New EPOC				
		missing (%)	*n* = 308 (%)	*n* = 153 (%)	*p-*value		308	153
Age at resection of CRLM - *(median [IQR])*			54.0 [46.0, 63.0]	64.0 [59.0, 69.0]	<0.001	0		
Gender	Male		185 (60)	104 (68)	0.10	0		
	Female		123 (40)	49 (32)				
Primary tumour location	Right-sided	21 (5)	77 (27)	30 (20)	0.06	4.6		
	Left-sided		124 (43)	61 (40)				
	Rectal		86 (30)	62 (41)				
T-stage	pT 0-2	24 (5)	47 (16)	16 (11)	0.15	5.2		
	pT 3-4		244 (84)	130 (89)				
N-stage	N0	24 (5)	94 (32)	47 (32)	0.96	5.2		
	*N* +		198 (68)	98 (68)				
Number of CRLM - *(median [IQR])*		6 (1)	2 [1,5]	2 [1,3]	<0.001	1.3		
Diameter of largest CRLM in cm - (median [IQR])		15 (3)	2.5 [1.7, 4.2]	1.9 [1.2, 3.3]	<0.001	3.3		
Disease-free interval in months^a^ - (median [IQR])		2 (0)	0 [0, 5]	1 [0, 13]	<0.001	0.4		
Preoperative CEA in µg/L - (median [IQR])		31 (7)	9.8 [3.8, 64.4]	12.9 [4.5, 34.0]	0.68	6.7		
Perioperative systemic chemotherapy	No	2 (0)	25 (8)	0 (0)	<0.001	0.4		
	Yes		281 (92)	153 (100)				
Resection margin involved	No	7 (2)	254 (83)	118 (79)	0.29	1.5		
	Yes		51 (17)	31 (21)				
Extrahepatic disease	No		236 (77)	149 (97)	<0.001	0		
	Yes		72 (23)	4 (3)				
Growth pattern phenotype	Non-encapsulated		246 (80)	111 (73)	0.08	0		
	Encapsulated		62 (20)	42 (27)				
Origin of sequenced sample	CRLM		157 (51)	142 (93)	<0.001	0		
	CRC		151 (49)	11 (7)				
Average coverage depth - (median [IQR])			714.0 [593.5, 843.5]	585.9 [477.2, 653.6]	<0.001	0		
Mutations per megabase - (median [IQR])			5.1 [3.6, 6.5]	6.1 [4.6, 9.1]	<0.001	0		

*CEA* carcinoembryonic antigen, *CRC* colorectal cancer, *CRLM* colorectal liver metastasis, *IQR* interquartile range, *MSKCC* Memorial Sloan Kettering Cancer Center.

^a^Between resection of primary tumour and detection of CRLM.

### Driver genes

**Fig. 1:**
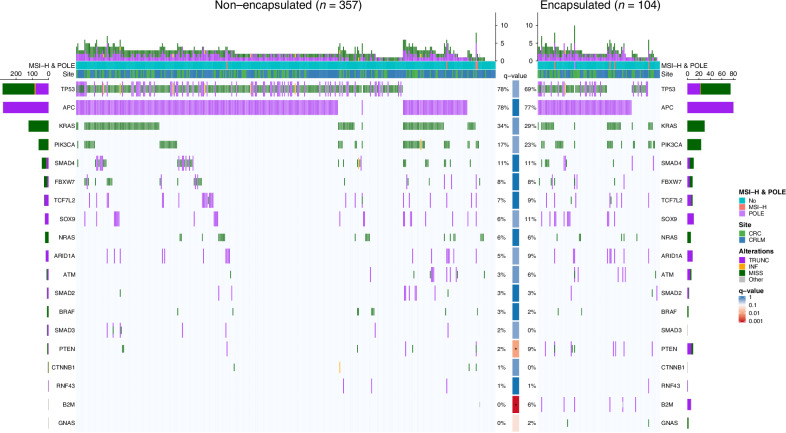
Comparison and graphical representation of the mutation rates of 19 colorectal cancer driver genes stratified by histopathological growth pattern and regarding microsatellite instability high and POLE mutant cases and genetic sample site (i.e., primay colorectal cancer or colorectal liver metastasis). The percentages represent the mutation frequency for each gene in each group. The q-value represents the result of the χ2 test with correction for multiple testing according to Benjamini & Hochberg applied. *q < 0.05. CRC colorectal cancer, CRLM colorectal liver metastasis, INF inframe, MISS missense, MSI-H microsatellite instability high, TRUNC truncating.

A total of 1116 (77%) SNV, 17 MNV (1%), and 312 (22%) indels were identified among the 19 driver genes of interest. Comparisons of the mutation rates of the 19 driver genes by growth pattern phenotype are reported and visualised in Fig. 1. The actionable colorectal cancer genes *KRAS, NRAS*, and *BRAF* were equally mutated in both phenotypes (q-values: 0.49, 0.97, and 0.97, respectively; Supplementary Table 2). After correcting for multiple testing, mutations in *B2M* (*q* = 0.001) and *PTEN* (*q* = 0.01) were associated with the encapsulated phenotype (Supplementary Table 2).

The *B2M* gene for beta-2-microglobulin showed pathogenic truncating mutations in 6% (*n* = 6/104) of encapsulated patients, whereas only a single (*n* = 1/357, 0.2%) start-loss mutation was discovered in non-encapsulated patients (*q* = 0.001, Fig. 1). When excluding MSI-H and *POLE* mediated hypermutation and after correction for TMB and cohort, the encapsulated phenotype remained independently associated with mutations in *B2M* (adjusted OR [95%CI]: 14.2 [1.5–131.4], Table 2), although the wide confidence interval suggests uncertainty in the estimate, likely due to the limited event numbers.

**Table 2:** Uni- and multivariable regression models

Logistic regression for B2M mutationsa^a^				
	Univariable		Multivariable (*n* = 452)	
	OR [95%CI]	*p*-value	OR [95%CI]	*p*-value
Encapsulated phenotype - yes vs no	14.82 [1.64-134.16]	0.020	14.18 [1.53−131.41]	0.02
Tumour mutational burden - cont.	1.04 [0.97-1.12]	0.270	1.02 [0.95−1.10]	0.61
Cohort - New EPOC vs MSKCC	1.32 [0.22-7.99]	0.76	0.98 [0.16-6.09]	0.98
Logistic regression for PTEN mutations^a^				
	Univariable		Multivariable (*n* = 452)	
	OR [95%CI]	*p*-value	OR [95%CI]	*p*-value
Encapsulated phenotype - yes vs no	5.30 [1.64-17.07]	0.005	4.85 [1.41-16.68]	0.01
Tumour mutational burden - cont.	1.11 [1.02-1.22]	0.020	1.18 [1.04-1.34]	0.009
Cohort - New EPOC vs MSKCC	0.39 [0.08-1.79]	0.22	0.14 [0.02-1.09]	0.06
Linear regression for TMB				
	Univariable		Multivariable (*n* = 461)	
	β [95%CI]	*p*-value	β [95%CI]	*p*-value
Encapsulated phenotype - yes vs no	4.10 [1.92; 6.27]	<0.001	n	0.01
MSI-H or POLE mutant - Yes vs No	50.88 [46.11; 55.65]	<0.001	50.57 [45.80; 55.34]	<0.001
Cohort - New EPOC vs MSKCC	0.82 [−1.14; 2.78]	0.410	1.61 [0.08; 3.15]	0.04
Sample site - CRC vs CRLM	0.75 [−1.19; 2.68]	0.450	−0.09 [-1.61; 1.42]	0.90

*CO* confidence interval, *cont.* entered as continous predictor, *CRC* colorectal cancer, *CRLM* colorectal liver metastasis, *MSI-H* microsatellite instability high, *OR* odds ratio, *TMB* tumour mutational burden.

^a^Excluding MSI-H or POLE mutant cancers (*n* = 9).

The phosphatase and tensin homologue (*PTEN)* gene was mutated in 9% (*n* = 9/104, 7 truncating 2 missense mutations) of the encapsulated group versus 2% (*n* = 7/357, 3 truncating, 4 missense mutations) of the non-encapsulated group (*q* = 0.01, Fig. 1). This association was independent of TMB and cohort when MSI-H and *POLE*-mutant tumours were excluded (adjusted OR [95%CI]: 4.9 [1.4–16.7], Table 2).

### Signalling pathways

The frequency of driver gene mutations belonging to the Wnt/β-catenin, MAPK, and TGF-β pathways did not differ by the growth pattern phenotype (all *p* > 0.4, Supplementary Fig. 2). Given the higher *PTEN* mutation rate in the encapsulated group, mutations in the PI3K pathway occurred more frequently in these patients (31% [32/104] vs. 18% [66/357], *p* = 0.007; Supplementary Fig. 2).

### Mutational load and hypermutation

**Fig. 2:**
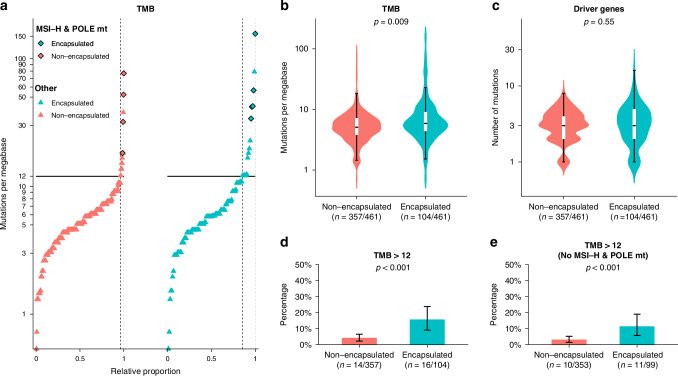
Tumour mutational burden and hypermutation frequency in encapsulated and non-encapsulated patients. **a** The distribution of tumour mutational burden is plotted for encapsulated and non-encapsulated patients with the number of mutations per megabase on the Y-axis (logarithmic scale), and the relative proportion of patients within the cohort on the X-axis. Each point represents a single patient. The horizontal line represents the cut-off for hypermutated forms of colorectal cancer (tumour mutational burden >12 mutations per megabase), and the dashed vertical line represents the intersection with this cut-off on the X-axis. **b** Violin and boxplots displaying the tumour mutational burden and (**c**) number of driver gene mutations on a logarithmic scale in encapsulated and non-encapsulated patients. The box and corresponding horizontal line represent the interquartile range and median, respectively, and the whiskers represent the range excluding outliers (defined according to the 1.5 rule). The *p*-value represents the result of the Mann–Whitney U test. **d** Barplots displaying the frequency of hypermutated tumours (defined as a tumour mutational burden greater than 12 mutations per megabase) with and (**e**) without microsatellite instability high and POLE mutant cases in encapsulated and non-encapsulated patients. The error bars represent the binomial 95% confidence interval according to Clopper-Pearson. The *p* value represents the result of the χ2 test. MSI-H microsatellite instability high, mt mutant.

Figure 2 shows the TMB, hypermutation, and number of driver gene mutations stratified by the growth pattern phenotype. TMB was significantly higher in the encapsulated phenotype with median (IQR) 5.8 (4.3–9.1) vs. 5.1 (3.6–7.2) mutations/Mb (*p* = 0.009, Fig. 2a, b), also independent of MSI-H and *POLE* hypermutability (adjusted β [95%CI]: 2.1 [0.5;3.7], Table 2). Hypermutation, defined as >12 mutations/Mb, was significantly more frequent in encapsulated samples (15% [16/104] vs. 4% [14/357], *p* < 0.001; Fig. 2d), as well as when excluding MSI-H and *POLE* mutant cases (11% [11/99] vs. 3% [10/353], *p* < 0.001; Fig. 2e). The number of driver gene mutations did not differ, with a median (IQR) of 3 (2–4) versus 3 (2–4) mutations in the 19 genes assessed for encapsulated versus non-encapsulated, respectively (*p* = 0.60, Fig. 2c).

### MSI-H and POLE mediated hypermutation

Eight MSI-H cancers and one pathogenic *POLE* mutation (V411L) were identified, which made up 30% of all hypermutated tumours and had a significantly higher TMB compared to the other samples; median 42.5 (34.0–56.5) versus 5.1 (3.9–7.2) mutations/Mb (*p* < 0.001), respectively. MSI-H and *POLE* mediated hypermutations were significantly more common in the encapsulated 5/104 (5%) than in the non-encapsulated 4/357 (1%) phenotype (*p* = 0.02, Supplementary table 1).

### Co-occurrence and mutual exclusivity

**Fig. 3:**
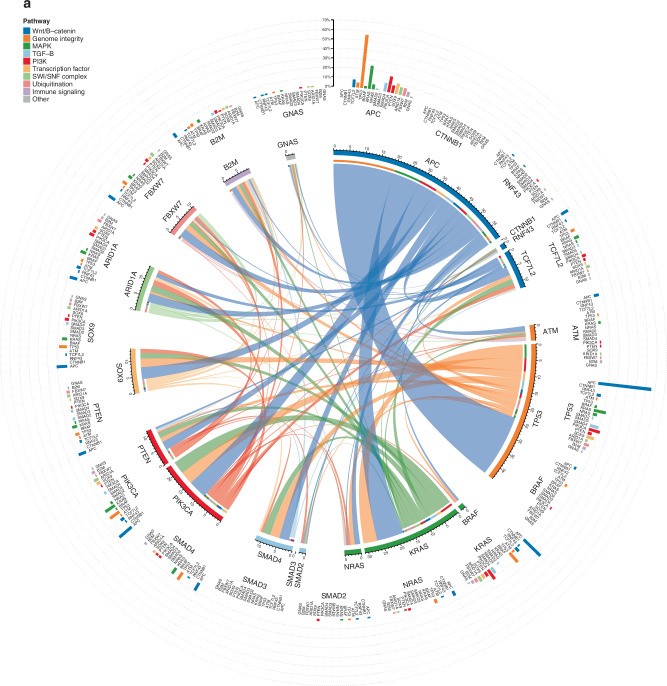

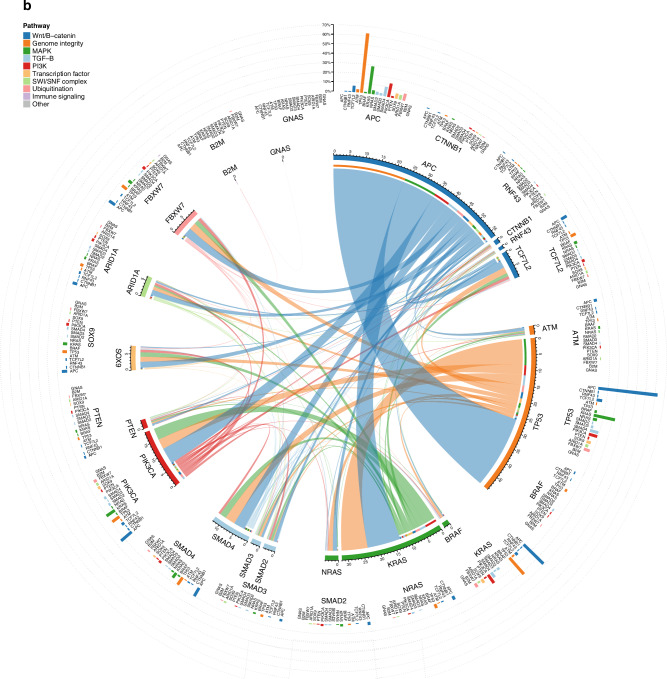
Co-occurence and double mutation rates of 19 driver genes in encapsualted and non-encapsulated patients. **a** Encapsulated and (**b**) non-encapsulated patient groups are shown, with genes grouped by genomic pathway. Within the inner circle, the frequency of co-occurring mutations between gene-pairs as a proportion of the total number of mutations is shown using ribbons, where the ruler indicates the proportion expressed as percentage; i.e., a percentage of 20 means that the co-occurring mutations in gene X and Y represented 20% of all mutations identified within the cohort. On the outer circle, the double mutation rates within the cohort for each gene pair is plotted using bar plots; i.e., a percentage of 20 means that in all patients, 20% had a mutation in gene X and a co-occurring mutation in gene Y.

Testing for mutual exclusivity in 357 non-encapsulated samples revealed *TP53* with *ATM/KRAS/PIK3CA* and *KRAS* with *NRAS* (q-values: 0.006, 0.001, <0.001, and <0.001, respectively), and *TP53* with *KRAS* (*q* = 0.008) in 104 encapsulated samples. Testing in all 461 samples with the growth pattern phenotype as a stratification factor again revealed *TP53* with *ATM/KRAS/PIK3CA* and *KRAS* with *NRAS* (all q-values < 0.001). Co-occurrence associations could not be demonstrated using the DISCOVER test. The relative co-occurrence and double-mutation rates of the driver genes are visualised for the encapsulated and non-encapsulated phenotypes in Fig. 3a, b, respectively.

### Survival

**Fig. 4:**
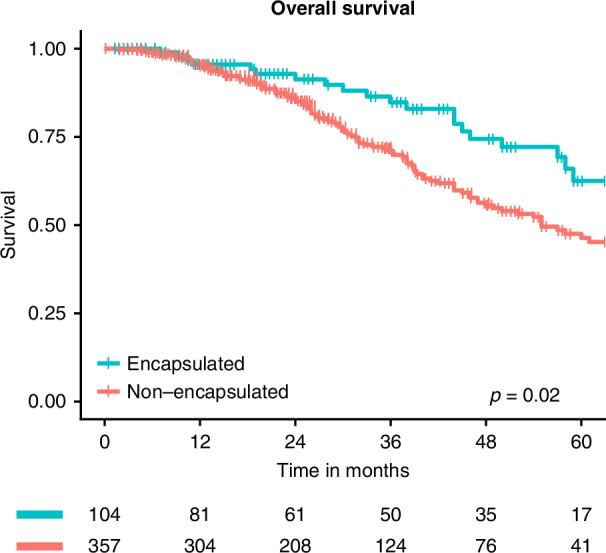
Kaplan–Meier overall survival estimates for encapsulated versus non-encapsulated patients after resection of colorectal liver metastasis. The *p* value represents the result of the overall log-rank test.

The median (IQR) follow-up period for survivors was 37 (20–59) months, during which 133 patients died. Survival was significantly longer for patients with an encapsulated phenotype, with 5-year (95%CI) estimates of 63% (50–79%) compared to 46% (39–55%) for non-encapsulated patients (*p* = 0.02, Fig. 4), and was independent of potential clinical and genetic confounders (adjusted HR [95%CI]: 0.60 [0.36–0.99], Supplementary Table 3).

### Stratified analyses

Comparisons of MSI-H and *POLE* mediated hypermutation and driver gene mutation frequencies are provided in Supplementary Table 4 and Supplementary Fig. 3 for MSKCC and 4 for New EPOC. Stratified analyses revealed that the associations identified were dependent on the MSKCC data. The associations between MSI-H and *POLE* mutant forms of colorectal cancer and *B2M* and *PTEN* mutations were only observed in MSKCC, with MSI-H and *POLE* mediated hypermutation occurring in 8% (5/62) vs. 1% (3/246) (*p* = 0.002), and mutation rates of 5% (8/62) vs. 0% (0/246) (*q* < 0.001) in *B2M* and 13% (8/62) vs. 2% (6/246) (*q* = 0.004) in *PTEN* for encapsulated versus non-encapsulated, respectively. In comparison, only one MSI-H tumour was identified in the New EPOC in a non-encapsulated patient (*p* = 0.54, Supplementary Table 4 and Supplementary Fig. 4), and for both *B2M* and *PTEN* only two pathogenic mutations were present, one in each growth pattern phenotype (both *q* = 0.80, Supplementary Table 4 and Supplementary Fig. 3). No significant associations between the growth pattern phenotype and genotype existed in either cohort for any of the other 17 driver genes investigated, except for *GNAS* in MSKCC, with mutation rates of 3% (2/62) vs. 0% (0/246) (*q* = 0.03) for encapsulated versus non-encapsulated, respectively (Supplementary Table 4). Similar findings were observed when comparing TMB, which was significantly higher (median [IQR]) for encapsulated patients in MSKCC (5.5 [4.3–6.5] vs. 4.6 [3.6–6.4] mutations/Mb, *p* = 0.02), but only showed a similar tendency in New EPOC (7.6 [4.6–10.7] vs. 6.1 [4.6–9.1] mutations/Mb, *p* = 0.39).

Considering any of the observed differences, 15% (16/104) of encapsulated patients had either MSI-H or *POLE* mediated hypermutation, or a mutation in *B2M* or *PTEN*, versus 3% (*n* = 10/357) of non-encapsulated patients (*p* < 0.001). However, with respective rates of 23% vs. 7% (*p* < 0.001) in MSKCC and 5% vs. 3% (*p* = 0.52) in New EPOC, this finding was also inconsistent between the cohorts.

### Driver gene mutations across origin of samples

Comparisons of MSI-H and *POLE* mediated hypermutation and driver gene mutation frequencies across sample origin are provided in Supplementary Fig. 5. Percentages of driver gene mutations were comparable between samples from CRLM and CRC origin.

## Discussion

In this study, we discovered through next-generation sequencing data from 461 patients across two cohorts that genetic heterogeneity between the growth pattern phenotypes is limited and mostly relates to genetic alterations (i.e., hypermutation) known to drive anticancer immunity.

In both the combined and stratified analyses, no apparent difference in tumorigenesis was revealed by the comparison of putative colorectal cancer driver genes between the growth pattern phenotypes. Encapsulated and non-encapsulated tumours were equally affected by *APC* and *TP53* loss, and oncogenic mutations of the MAP kinase and TGF-β pathways, all known hallmarks of colorectal carcinogenesis [33]. Specifically with regard to the current markers for EGFR inhibition therapy and known (metastatic) colorectal cancer risk factor genes *KRAS*, *NRAS*, and *BRAF*, no association with growth patterns was found [34–36]. Herein, it is important to note that the mutation rate of several of these genes in the New EPOC cohort is not representative of the real-world population of patients with liver-metastatic CRC, as the wild-type status of *KRAS* exon-2 (codons 12, 13, and 61) was a prerequisite for trial eligibility, posing a limitation to our study. Nevertheless, these results, including our analysis of overall survival, support the previous observation that the survival difference between the growth pattern phenotypes is separate from the prognostic impact of *KRAS* and *BRAF* mutations [21]. When considering all (potential) elements of genetic heterogeneity identified (i.e., MSI-H and *POLE* mediated hypermutation, and *B2M* or *PTEN* mutations), the majority of encapsulated patients (i.e., 85% in total, 77% in MSKCC, and 95% for New EPOC) exhibited none of these traits, and were essentially equally affected as non-encapsulated patients by (un)favourable genetic risk factors. Therefore, this study did not identify oncogenetics, at least not at the DNA level, as the major mechanism responsible for the growth pattern phenotypes of colorectal liver metastases [37, 38].

In this study we have accounted for tumour heterogeneity despite challenges in methodological approaches. Firstly, there is no significant intra- and intermetastasis heterogeneity regarding growth pattern, as we demonstrated previously [25]. Heterogeneity in genomic profile between and within metastases may exist on some level, however previous research investigating this has shown high intrapatient concordance for common driver gene mutations [39, 40]. This is in line with the comparison of driver gene mutations between primary tumour and metastases in the current cohort, which showed no significant statistical difference.

Hypermutation, both independent of and related to MSI-H or *POLE* mutant forms of colorectal cancer, was more common in patients with encapsulated liver metastases. It is the current belief that the greater the number of mutations, the higher the probability of immunogenic variants, meaning potential effective targets for immune response [5, 41]. This is especially true for clonal mutations developing early on in the tumorigenesis (i.e., oncogenic drivers) rather than those arising later on, remaining limited to smaller tumour cell subpopulations [42]. This preponderance for hypermutated tumours suggests that genetically driven adaptive T-cell responses may be more prevalent in the encapsulated phenotype. With only one MSI-H tumour observed in the New EPOC, the numbers were insufficient to reliably assess this association in the stratified analysis, complicating the interpretation of these results.

Similar to *B2M*, mutations in the ‘phosphatase-and-tensin-homologue’ *PTEN* tumour suppressor gene are more frequently found in MSI-H colorectal cancer, and specifically in locally advanced or metastatic cancers [43]. While mutations in *PTEN* generally lead to downregulation, silencing is also known to occur in colorectal cancer through epigenetic inactivation [43]. We found that mutations in *PTEN* are associated with a encapsulated phenotype, but again with conflicting results between cohorts. Additionally, epigenetic forms of silencing were not considered in this study. Low *PTEN* expression has been associated with inferior survival outcomes after resection of colorectal liver metastasis [44]. It therefore, it seems counterintuitive that *PTEN* mutations would be increased in patients with a encapsulated phenotype, given their superior survival [20, 21]. *PTEN* expression and/or protein levels should therefore be considered in the potential association between *PTEN* silencing and growth pattern phenotypes. This seems all the more relevant given the emerging role of *PTEN* in evasion of the immune response across several tumour types, including melanoma and glioblastoma where *PTEN* loss has been associated with reduced response to immunotherapy [45–47]. However, reports are also conflicting across tumour types, as *PTEN* loss has been associated with both pro-inflammatory mechanisms through its role in DNA repair defects with deficient tumours having higher genomic instability leading to increased neoantigens and a higher probability of immune response, but also anti-inflammatory mechanisms through increased infiltration of T regulatory cells, myeloid-derived suppressor cells (MDSC), tumour associated macrophages, and increased PD-L1 expression [45]. Specifically for colorectal cancer, *PTEN* loss has been linked with increased expression of PD-L1 and expansion of tumour-associated MDSC [48, 49]. Given there are currently two trials underway directly targeting *PTEN* deficiency (NCT01884285, NCT01458067), and in-vitro models have shown the combination of immunotherapy and PI3Kβ inhibition to increase response, *PTEN* may become an actionable target in the future and revisiting the potential association between the growth patterns and *PTEN* mutations may therefore be considered [46].

Several limitations of the present study should be acknowledged. Firstly, the inherent differences of the two patient cohorts, in addition to the differences in the origin of samples as discussed previously the median age differed between the two cohorts, namely, 57 years in the MSKCC cohort vs. 67 years in the New EPOC cohort. This is likely due to a referral bias as the MSKCC is a single (inter)national expert centre, and the New EPOC study is performed in multiple smaller hospitals. Another limitation of the study is the use of targeted NGS rather than whole-genome sequencing, which restricts analysis to predefined genomic regions. Lastly, the high prevalence of pre-operative chemotherapy in both cohorts may influence the outcome of this study as several studies have shown that the prognostic value of the growth patterns is diminished after chemotherapy treatment.

In conclusion, results in and across both cohorts do not find evidence for a major difference in gene alterations identified at the DNA level and consequently point to biological mechanisms other than oncogenetics underlying the prognostic impact of these histologic phenotypes, epigenetic transcriptional reprogramming being one of the obvious explanatory mechanisms [50]. While associations between genetic drivers of adaptive anti-cancer immunity and the encapsulated growth pattern were observed and could potentially explain the inflamed status of a minority of the metastases, results were conflicting between cohorts and require additional research.

## Supplementary information

Supplementary material HGP mutations

## Data Availability

The data analyzed in this study are available from the MSKCC and New EPOC trials. Restrictions apply to the availability of these data, which were used under license in these studies. Data are available from the authors upon reasonable request with permission from the MSKCC or New EPOC.

## Code availability

Code is available upon request and at discretion of the senior author of the paper.
