# Supplementary material for: The colorectal liver metastasis growth pattern phenotype is not dependent on genotype
Source: Br J Cancer. 2025 Jul 23;133(7):945–55. doi: 10.1038/s41416-025-03103-4 (PMC12480539; doi:10.1038/s41416-025-03103-4)
Supplement: Supplementary file 1 — Supplementary material HGP mutations [file 41416_2025_3103_MOESM1_ESM.pdf]

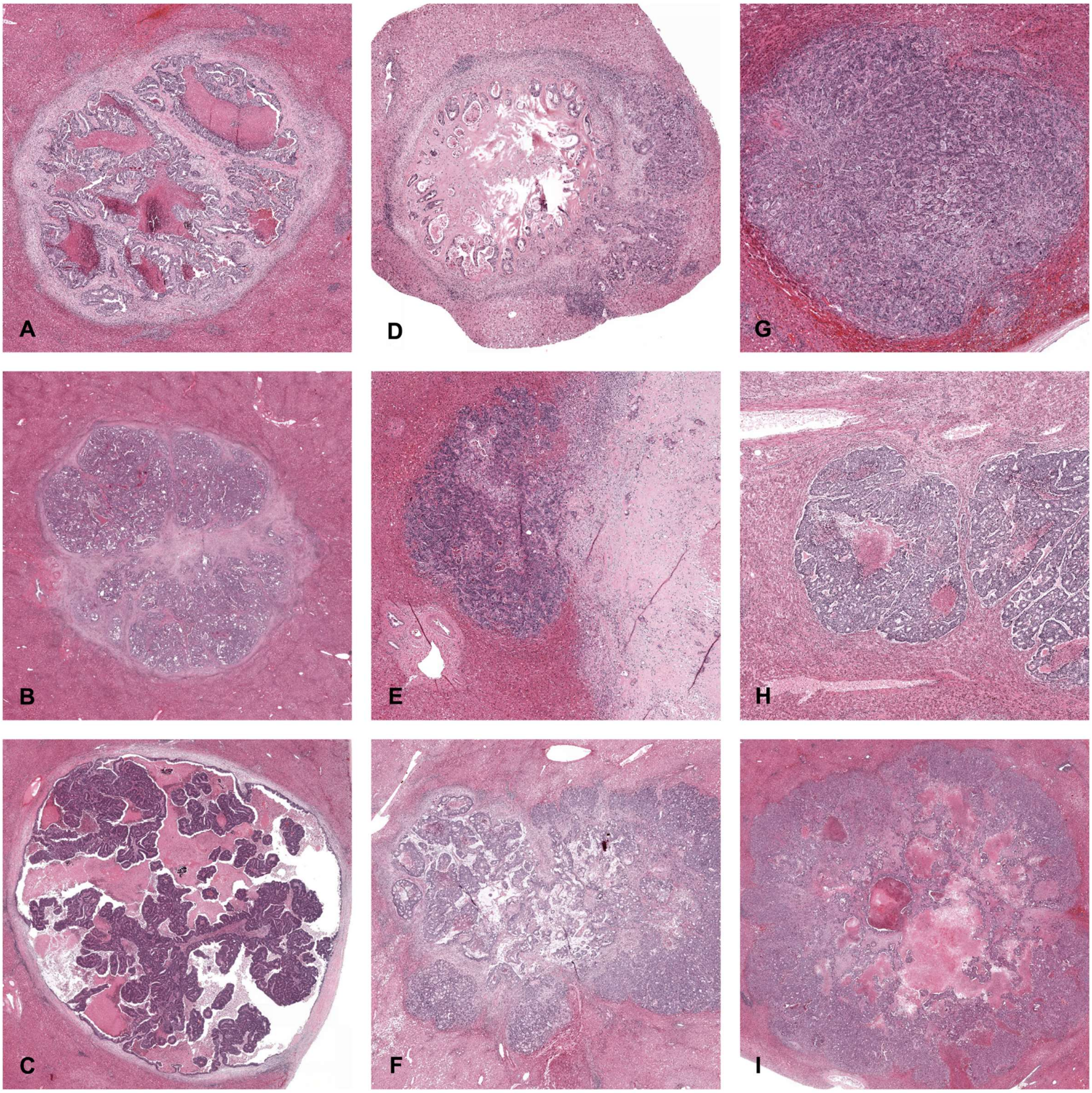

Supplementary figure 1. H&E examples of desmoplastic (A-C) and non-desmoplastic (D-I) colorectal cancer liver metastases.

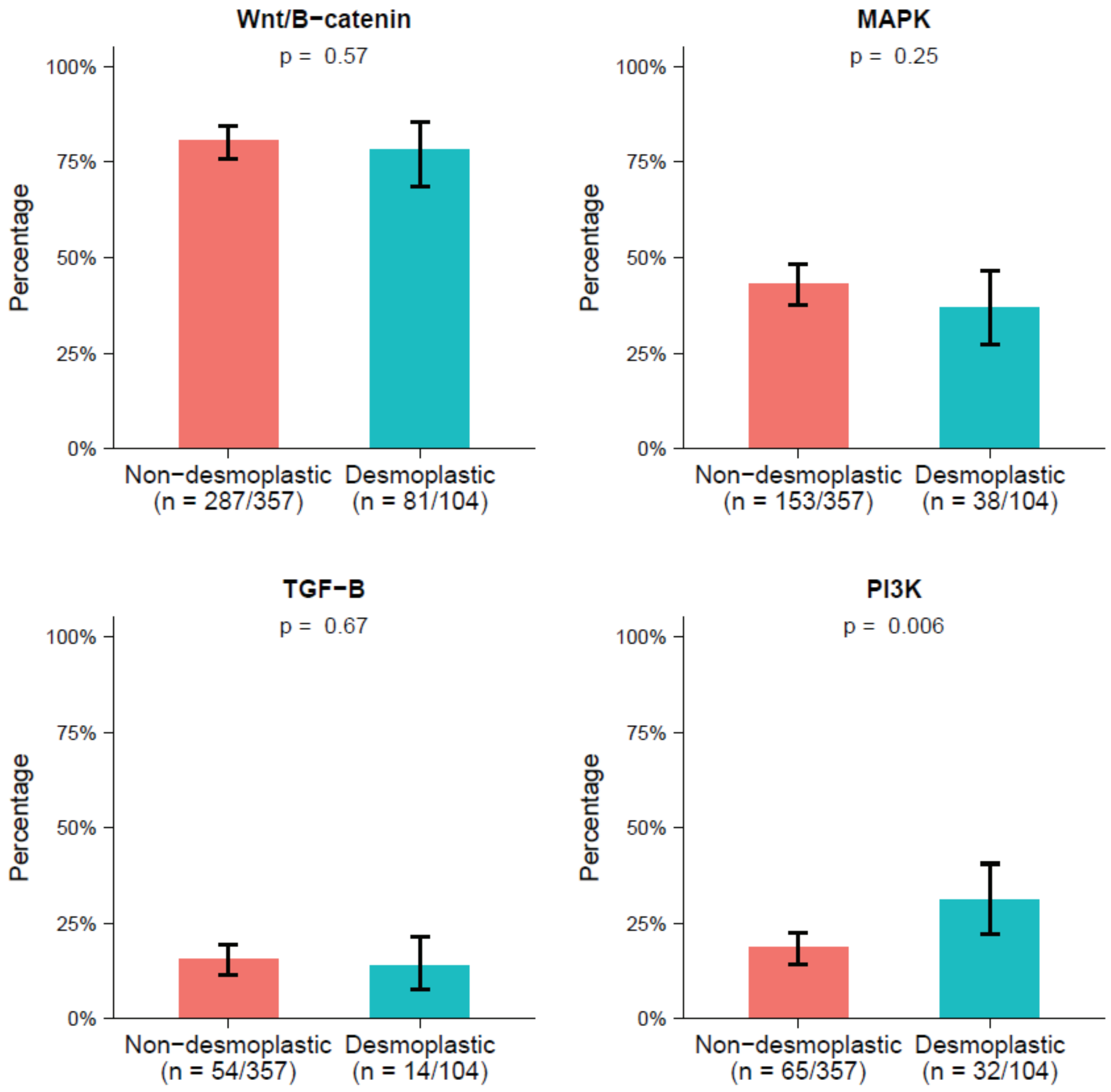

Supplementary figure 2. Bar plots representing the frequency of driver gene mutations in percentage belonging to Wnt/Beta-catenin, MAPK, TGF-beta, and PI3K pathway by histopathological growth pattern. The error bars represent the binomial 95% confidence interval according to Clopper-Pearson. The p-value represents the result of the $\chi 2$ test.

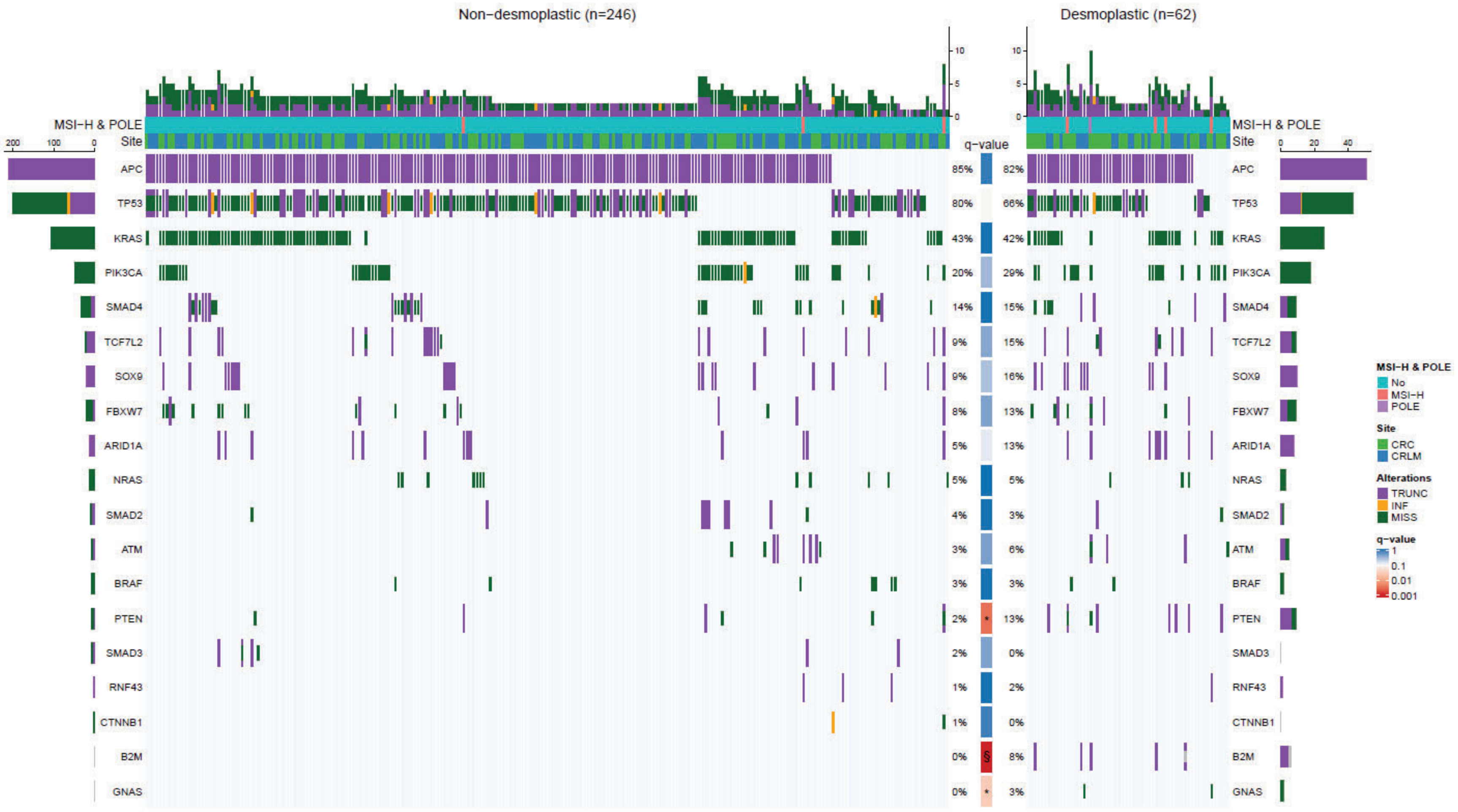

Supplementary figure 3. Comparison and graphical representation of the mutation rates of 19 colorectal cancer driver genes in the **Memorial Sloan Kettering Cancer Center (MSKCC) cohort** stratified by histopathological growth pattern and in regards to microsatellite instability high and POLE mutant cases and genetic sample site (i.e. primay colorectal cancer or colorectal liver metastasis). The percentages represent the mutation frequency for each gene in each group. The q-value represents the result of the $\chi2$ test with correction for multiple testing according to Benjamini & Hochberg applied. * $q < 0.05$. CRC = colorectal cancer; CRLM = colorectal liver metastasis, INF = inframe, MISS = missense, MSI-H = microsatellite instability high, TRUNC = truncating.

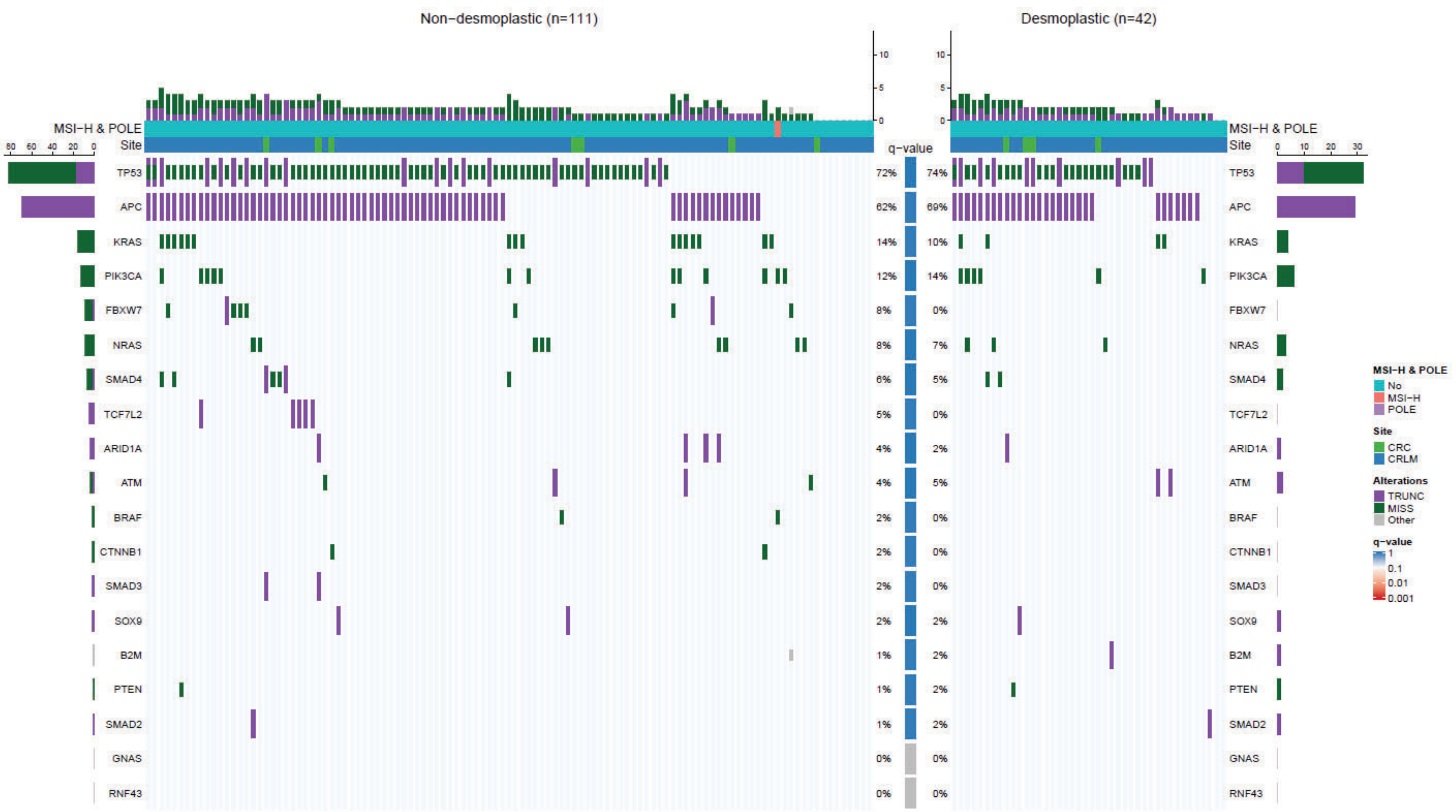

Supplementary figure 4. Comparison and graphical representation of the mutation rates of 19 colorectal cancer driver genes in the **New-EPOC trial cohort** stratified by histopathological growth pattern and in regards to microsatellite instability high and POLE mutant cases and genetic sample site (i.e. primay colorectal cancer or colorectal liver metastasis). The percentages represent the mutation frequency for each gene in each group. The q-value represents the result of the χ2 test with correction for multiple testing according to Benjamini & Hochberg applied. * q < 0.05. CRC = colorectal cancer; CRLM = colorectal liver metastasis, INF = inframe, MISS = missense, MSI-H = microsatellite instability high, TRUNC = truncating.

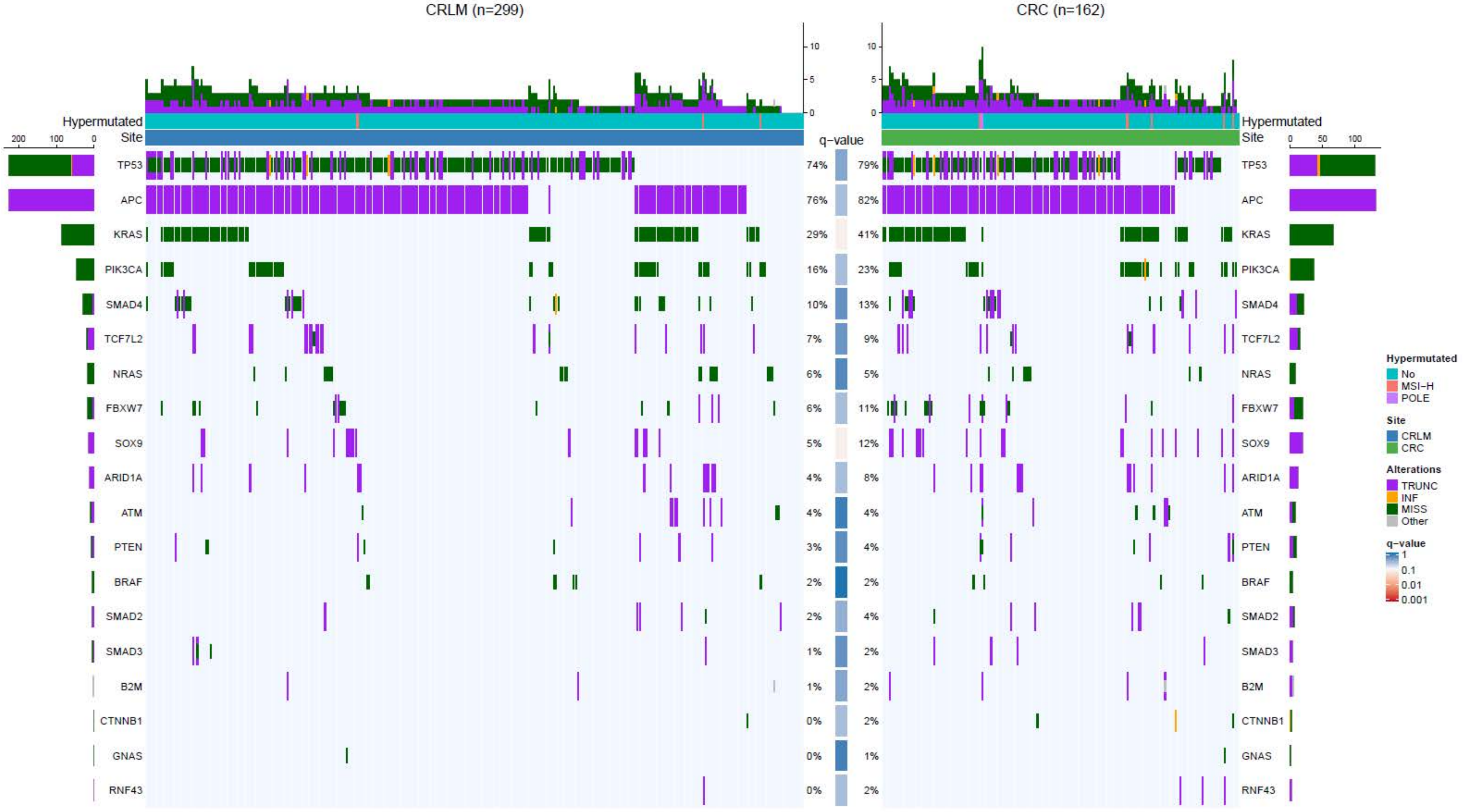

Supplementary figure 5. Comparison and graphical representation of the mutation rates of 19 colorectal cancer driver genes in the combined cohort stratified by genetic sample site and in regards to microsatellite instability high and POLE mutant cases. The percentages represent the mutation frequency for each gene in each group. The q-value represents the result of the χ2 test with correction for multiple testing according to Benjamini & Hochberg applied. * q < 0.05. CRC = colorectal cancer; CRLM = colorectal liver metastasis, INF = inframe, MISS = missense, MSI-H = microsatellite instability high, TRUNC = truncating.

Supplementary table 1. Next generation sequencing panels

Panel	Genes
IMPACT-410	ABL1, ACVR1, AKT1, AKT2, AKT3, ALK, ALOX12B, ANKRD11, APC, AR, ARAF, ARID1A, ARID1B, ARID2, ARID5B, ASXL1, ASXL2, ATM, ATR, ATRX, AURKA, AURKB, AXIN1, AXIN2, AXL, B2M, BAP1, BARD1, BBC3, BCL10, BCL2, BCL2L1, BCL2L11, BCL6, BCOR, BIRC3, BLM, BMPR1A, BRAF, BRCA1, BRCA2, BRD4, BRIP1, BTK, CALR, CARD11, CASP8, CBFB, CBL, CCND1, CCND2, CCND3, CCNE1, CD274, CD276, CD79A, CD79B, CDC73, CDH1, CDK12, CDK4, CDK6, CDK8, CDKN1A, CDKN1B, CDKN2A, CDKN2B, CDKN2C, CEBPA, CENPA, CHEK1, CHEK2, CIC, CREBBP, CRKL, CRLF2, CSF1R, CSF3R, CTCF, CTLA4, CTNNB1, CUL3, CXCR4, DAXX, DCUN1D1, DDR2, DICER1, DIS3, DNAJB1, DNMT1, DNMT3A, DNMT3B, DOT1L, E2F3, EED, EGFL7, EGFR, EIF1AX, EIF4A2, EIF4E, EP300, EPCAM, EPHA3, EPHA5, EPHA7, EPHB1, ERBB2, ERBB3, ERBB4, ERCC2, ERCC3, ERCC4, ERCC5, ERG, ERRFI1, ESR1, ETV1, ETV6, EZH2, FAM123B, FAM175A, FAM46C, FANCA, FANCC, FAT1, FBXW7, FGF19, FGF3, FGF4, FGFR1, FGFR2, FGFR3, FGFR4, FH, FLCN, FLT1, FLT3, FLT4, FOXA1, FOXL2, FOXO1, FOXP1, FUBP1, FYN, GATA1, GATA2, GATA3, GLI1, GNA11, GNAQ, GNAS, GPS2, GREM1, GRIN2A, GSK3B, H3F3A, H3F3B, H3F3C, HGF, HIST1H1C, HIST1H2BD, HIST1H3A, HIST1H3B, HIST1H3C, HIST1H3D, HIST1H3E, HIST1H3F, HIST1H3G, HIST1H3H, HIST1H3I, HIST1H3J, HIST2H3C, HIST2H3D, HIST3H3, HLA-A, HNF1A, HOXB13, HRAS, ICOSLG, ID3, IDH1, IDH2, IFNGR1, IGF1, IGF1R, IGF2, IKBKE, IKZF1, IL10, IL7R, INHA, INHBA, INPP4A, INPP4B, INSR, IRF4, IRS1, IRS2, JAK1, JAK2, JAK3, JUN, KDM5A, KDM5C, KDM6A, KDR, KEAP1, KIT, KLF4, KRAS, LATS1, LATS2, LMO1, MALT1, MAP2K1, MAP2K2, MAP2K4, MAP3K1, MAP3K13, MAP3K14, MAPK1, MAPK3, MAX, MCL1, MDC1, MDM2, MDM4, MED12, MEF2B, MEN1, MET, MGA, MITF, MLH1, MLL, MLL2, MLL3, MPL, MRE11A, MSH2, MSH6, MST1, MST1R, MTOR, MUTYH, MYC, MYCL1, MYCN, MYD88, MYOD1, NBN, NCOA3, NCOR1, NEGR1, NF1, NF2, NFE2L2, NFKBIA, NKX2-1, NKX3-1, NOTCH1, NOTCH2, NOTCH3, NOTCH4, NPM1, NRAS, NSD1, NTRK1, NTRK2, NTRK3, NUP93, PAK1, PAK7, PALB2, PARK2, PARP1, PAX5, PBRM1, PDCD1, PDGFRA, PDGFRB, PDPK1, PGR, PHOX2B, PIK3C2G, PIK3C3, PIK3CA, PIK3CB, PIK3CD, PIK3CG, PIK3R1, PIK3R2, PIK3R3, PIM1, PLCG2, PLK2, PMAIP1, PMS1, PMS2, PNRC1, POLD1, POLE, PPM1D, PPP2R1A, PPP6C, PRDM1, PRKAR1A, PTCH1, PTEN, PTPN11, PTPRD, PTPRS, PTPRT, RAB35, RAC1, RAD21, RAD50, RAD51, RAD51C, RAD51L1, RAD51L3, RAD52, RAD54L, RAF1, RARA, RASA1, RB1, RBM10, RECQL4, REL, RET, RFWD2, RHEB, RHOA, RICTOR, RIT1, RNF43, ROS1, RPS6KA4, RPS6KB2, RPTOR, RUNX1, RYBP, SDHA, SDHAF2, SDHB, SDHC, SDHD, SETD2, SF3B1, SH2B3, SH2D1A, SHQ1, SMAD2, SMAD3, SMAD4, SMARCA4, SMARCB1, SMARCD1, SMO, SOCS1, SOX17, SOX2, SOX9, SPEN, SPOP, SRC, SRSF2, STAG2, STAT3, STAT5A, STAT5B, STK11, STK40, SUFU, SUZ12, SYK, TBX3, TCEB1, TCF3, TCF7L2, TERT, TET1, TET2, TGFBR1, TGFBR2, TMEM127, TMPRSS2, TNFAIP3, TNFRSF14, TOP1, TP53, TP63, TRAF2, TRAF7, TSC1, TSC2, TSHR, U2AF1, VEGFA, VHL, VTCN1, WT1, XIAP, XPO1, XRCC2, YAP1, YES1, ZFHX3, ZRSR2
IMPACT-468 (additional genes to IMPACT-410 panel)	AGO2, BABAM1, CARM1, CDC42, CSDE1, CYLD, CYSLTR2, DROSHA, DUSP4, ELF3, EPAS1, ERF, EZH1, FAM58A, HLA-B, INPPL1, KMT2B, KMT5A, KNSTRN, LYN, MAPKAP1, MSH3, MSI1, MSI2, NTHL1, NUF2, PDCD1LG2, PPARG, PPP4R2, PRDM14, PREX2, PRKCI, PRKD1, PTP4A1, RAC2, RECQL, RRAGC, RRAS, RRAS2, RTEL1, RXRA, SESN1, SESN2, SESN3, SHOC2, SLX4, SMYD3, SOS1, SPRED1, STK19, TAP1, TAP2, TEK, TP53BP1, UPF1, WHSC1, WHSC1L1, WWTR1
S:CORT	ACVR1B, ACVR2A, AKT1, AMER1, APC, ARID1A, ATM, ATP1B4, ATR, AXIN2, B2M, BCL9, BCL9L, BMPR2, BRAF, BUB1B, CASP8, CD58, CDC27, CDK8, CDKN2A, CDX2, CREBBP, CTNNB1, ELF3, EP300, ERBB2, ERBB3, FAM123B, FBXW7, FGFR3, FLT3, GNAS, HDLBP, HLA-A, HLA-B, HLA-C, HNF4A, HRAS, IDH1, IGF2, IKBKB, KAT6A, IRS2, KRAS, LIFR, MAP2K4, MBD6, MET, MLH1, MSH2, MSH3, MSH6, MYC, NF1, NRAS, NUGGC, PCBP1, PIK3CA, PIK3R1, PMS2, POLE, PPP2R1A, PTEN, RAF1, RBM10, RNF43, SEMG2, SMAD2, SMAD3, SMAD4, SMARCA4, SOX9, TCF7L2, TGIF1, TP53, UBR5, WBP1, ZFP36L2, ZNF781

Supplementary table 2. Comparison of MSI-H and *POLE* mediated hypermutation and mutation rates in 19 CRC driver

	Non-encapsulated n = 357 (%)	Encapsulated n = 104 (%)	q-value*
MSI-H or POLE-mt	4 (1)	5 (5)	p=0.02
APC	279 (78)	80 (77)	0,97
ARID1A	17 (5)	9 (9)	0,39
ATM	12 (3)	6 (6)	0,49
B2M	1 (0)	6 (6)	0,001
BRAF	9 (3)	2 (2)	0,97
CTNNB1	4 (1)	0 (0)	0,49
FBXW7	28 (8)	8 (8)	0,97
GNAS	0 (0)	2 (2)	0,05
KRAS	123 (34)	30 (29)	0,49
NRAS	21 (6)	6 (6)	0,97
PIK3CA	61 (17)	24 (23)	0,39
PTEN	7 (2)	9 (9)	0,01
RNF43	3 (1)	1 (1)	0,97
SMAD2	10 (3)	3 (3)	0,97
SMAD3	8 (2)	0 (0)	0,39
SMAD4	41 (11)	11 (11)	0,97
SOX9	23 (6)	11 (11)	0,39
TCFL2	26 (7)	9 (9)	0,97
TP53	277 (78)	72 (69)	0,38

**Comparisons of mutation rates was performed using the* χ^2 *test with correction for multiple testing according to Benjamini & Hochberg.*
CRC: colorectal cancer; MSI-H: microsatellite instability high; mt: mutant.

Supplementary table 3. Cox proportional hazards regression analysis for overall survival.

	Overall survival (n=432)			
	Univariable		Multivariable	
	HR [95%CI]	p-value	HR [95%CI]	p-value
Cohort - *New EPOC vs MSKCC*	0.81 [0.55-1.20]	0,29	1.33 [0.82-2.15]	0,25
N-stage - *N+ vs N0*	1.67 [1.10-2.53]	0,02	1.61 [1.05-2.48]	0,03
Number of CRLM - *>1 vs 1*	1.03 [0.73-1.47]	0,85	1.12 [0.77-1.64]	0,55
Extrahepatic disease - *yes vs no*	2.44 [1.54-3.86]	<0.001	1.95 [1.11-3.42]	0,02
MSI-H or *POLE* mutant - *yes vs no*	0.43 [0.06-2.97]	0,39	0.63 [0.06-6.21]	0,69
KRAS - *mt vs wt*	2.03 [1.40-2.95]	<0.001	2.31 [1.44-3.69]	<0.001
NRAS - *mt vs wt*	1.68 [0.97-2.93]	0,07	2.08 [1.12-3.83]	0,02
BRAF - *mt vs wt*	1.97 [0.73-5.35]	0,18	1.49 [0.31-7.13]	0,62
APC - *mt vs wt*	0.82 [0.57-1.18]	0,28	1.01 [0.66-1.56]	0,95
TP53 - *mt vs wt*	0.82 [0.56-1.20]	0,30	0.88 [0.56-1.38]	0,58
B2M - *mt vs wt*	0.52 [0.07-3.68]	0,51	1.01 [0.13-8.00]	0,99
PTEN - *mt vs wt*	0.87 [0.28-2.73]	0,81	0.64 [0.15-2.81]	0,55
Encapsulated phenotype - *yes vs no*	0.57 [0.36-0.91]	0,02	0.60 [0.36-0.99]	0,046

CI: confidence interval; CRLM: colorectal liver metastasis; HR: hazard ratio; MSI-H: microsatellite instability high; mt: mutant; MSKCC: Memorial Sloan Kettering Cancer Center; wt: wildtype.

Supplementary table 4. Stratified comparison of MSI-H and *POLE* mediated hypermutation and mutations in 19 CRC driver genes stratified by cohort

	MSKCC			New EPOC		
	Non-desmoplastic n = 246 (%)	Desmoplastic n = 62 (%)	q-value*	Non-desmoplastic n = 111 (%)	Desmoplastic n = 42 (%)	q-value*
MSI-H or POLE mt	3 (1)	5 (8)	p=0.002	1 (1)	0 (0)	p=0.54
APC	210 (85)	51 (82)	0.79	69 (62)	29 (69)	0.80
ARID1A	13 (5)	8 (13)	0.13	4 (4)	1 (2)	0.84
ATM	8 (3)	4 (6)	0.42	4 (4)	2 (5)	0.84
B2M	0 (0)	5 (8)	<0.001	1 (1)	1 (2)	0.802
BRAF	7 (3)	2 (3)	0.94	2 (2)	0 (0)	0.80
CTNNB1	2 (1)	0 (0)	0.75	2 (2)	0 (0)	0.80
FBXW7	19 (8)	8 (13)	0.41	9 (8)	0 (0)	0.80
GNAS	0 (0)	2 (3)	0.03	0 (0)	0 (0)	-
KRAS	107 (43)	26 (42)	0.94	16 (14)	4 (10)	0.80
NRAS	12 (5)	3 (5)	0.99	9 (8)	3 (7)	0.84
PIK3CA	48 (20)	18 (29)	0.28	13 (12)	6 (14)	0.84
PTEN	6 (2)	8 (13)	0.004	1 (1)	1 (2)	0.80
RNF43	3 (1)	1 (2)	0.94	0 (0)	0 (0)	-
SMAD2	9 (4)	2 (3)	0.94	1 (1)	1 (2)	0.80
SMAD3	6 (2)	0 (0)	0.41	2 (2)	0 (0)	0.80
SMAD4	34 (14)	9 (15)	0.94	7 (6)	2 (5)	0.84
SOX9	21 (9)	10 (16)	0.24	2 (2)	1 (2)	0.84
TCFL2	21 (9)	9 (15)	0.37	5 (5)	0 (0)	0.80
TP53	197 (80)	41 (66)	0.09	80 (72)	31 (74)	0.84

**Comparisons of mutation rates was performed using the* χ^2 *test with correction for multiple testing according to Benjamini & Hochberg.*

CRC: colorectal cancer; MSI-H: microsatellite instability high; mt: mutant.
